# Injectable Tannin-Containing Hydroxypropyl Chitin Hydrogel as Novel Bioactive Pulp Capping Material Accelerates Repair of Inflamed Dental Pulp

**DOI:** 10.3390/biom14091129

**Published:** 2024-09-06

**Authors:** Linfang Zhou, Wenjie Shi, Xinye Zhang, Ming Liu, Lu Zhang, Xulin Jiang, Zhi Chen

**Affiliations:** 1 State Key Laboratory of Oral & Maxillofacial Reconstruction and Regeneration, Key Laboratory of Oral Biomedicine Ministry of Education, Hubei Key Laboratory of Somatology, School & Hospital of Stomatology, Wuhan University, Wuhan 430079, China; zhoulf@whu.edu.cn (L.Z.); xinyezhang98@whu.edu.cn (X.Z.); mingliu_dentist@whu.edu.cn (M.L.); luzhang2012@whu.edu.cn (L.Z.); 2 Key Laboratory of Biomedical Polymers of Ministry of Education & Department of Chemistry, Wuhan University, Wuhan 430072, China; shiwenjie@whu.edu.cn

**Keywords:** pulpitis, vital pulp therapy, pulp capping material, tannic acid, injectable hydrogel

## Abstract

Conventional pulp capping materials have limited anti-inflammatory capacity. It is necessary to develop more effective pulp capping material for the treatment of inflamed pulps. Tannic acid (TA) is a natural, water-soluble polyphenol with antimicrobial and anti-inflammatory properties. This study aimed to investigate the effects of a tannin-containing hydroxypropyl chitin hydrogel (HPCH/TA hydrogel) as an innovative pulp capping material. The physicochemical properties of the composite hydrogels were characterized. The effects of HPCH/TA hydrogel as a pulp capping material were evaluated in vitro and in vivo. The underlying mechanism of the anti-inflammatory effects of HPCH/TA hydrogel was explored. The HPCH/TA hydrogel demonstrated favorable temperature sensitivity, injectability, and antibacterial properties. In vitro, the HPCH/TA hydrogel effectively promoted the proliferation of human dental pulp cells and inhibited interleukin-1β, interleukin-6, and tumor necrosis factor-α expression, possibly by suppressing the nuclear factor kappa-B pathway. In vivo, on the fourth day after capping, the HPCH/TA hydrogel group showed lower inflammatory scores compared to the control and iRoot BP Plus (commercial pulp capping material) group. By the sixth week, complete reparative dentin formation was observed in the HPCH/TA hydrogel group, with no difference in thickness compared to the iRoot BP Plus group. Collectively, the HPCH/TA hydrogel holds promise as a bioactive pulp capping material for promoting the repair of inflamed pulp in vital pulp therapy.

## 1. Introduction

The dental pulp plays a crucial role in nourishing, sensing, defending, and repairing the tooth. Infection of the pulp can lead to infectious inflammation, which may result in pain, pulp necrosis, and abscess formation [1]. Pulpitis is characterized by spontaneous, impulsive, and nocturnal pain. The main treatment for pulpitis is root canal therapy (RCT), which aims to preserve the affected tooth by removing the infected pulp [2]. However, after pulp removal, the sensory function, the immune defense mechanism, and the self-repair capacity of the affected teeth are lost [3]. In addition, from a clinical perspective, teeth with vital pulp have greater resistance to biting forces than teeth that have undergone RCT, thereby reducing the risk of root fracture [4]. Therefore, vital pulp is critical to the longevity of teeth.

The goal of vital pulp therapy (VPT) is to preserve the vitality and function of the dental pulp after trauma or caries [5]. VPT procedures include indirect or direct pulp capping, as well as partial or complete pulpotomy [6]. Calcium hydroxide was previously used as the “gold standard” for pulp capping, but it has drawbacks such as inflammation and necrosis of the pulp surface after pulp capping [7,8]. Calcium silicate-based cements (CSCs), such as mineral trioxide aggregate (MTA), are commonly used for VPT. However, CSCs have drawbacks such as a long setting time, inconvenience in handling, and the potential for tooth discoloration and pulp tissue necrosis [9,10]. Moreover, calcium silicate-based materials have only limited anti-inflammatory properties [10]. If the pulp capping material exhibits anti-inflammatory properties, it has the potential to enhance the success rate of VPT in inflamed pulps. It is necessary to develop more effective pulp capping material for the treatment of inflamed pulps [11].

Hydrogel is a material that consists of a three-dimensional hydrophilic network with high water content. Its structure is similar to the natural extracellular matrix, making it useful for various biomedical applications, such as molecule and cell delivery and tissue regeneration and repair [12]. Several studies [13,14,15,16,17] have confirmed the suitability of hydrogels as an ideal vehicle for pulp capping materials. For instance, Zhu et al. [17] demonstrated that Silver-Doped Bioactive Glass/Chitosan Hydrogel can promote affected tissue recovery and increase the pulp tissue retention rate of pulp-affected immature permanent teeth. Furthermore, Holiel et al. [16] discovered that dentin matrix hydrogel facilitates dentin regeneration and preserves pulp vitality, potentially serving as a viable natural alternative to silicate-based cements. Chitin, the most abundant amino polysaccharide in nature, demonstrates good biocompatibility and bioactivity [18]. According to Yuan et al. [19], hydroxypropyl chitin (HPCH) can be synthesized from chitin via etherification with propylene oxide in NaOH/urea solvent. HPCH exhibits reversible thermosensitivity, quickly transforming into a gel under physiological conditions without the need for additional chemicals. Furthermore, HPCH can efficiently carry molecules and facilitate slow molecule release [19].

Tannic acid (TA) is a natural, water-soluble polyphenol that is widely distributed in plants and available from a variety of sources [20]. TA is highly biocompatible and has been approved as a food additive by the US Food and Drug Administration [21]. TA exhibits extensive physiological effects, such as antioxidant, antitumor, antimicrobial, and anti-inflammatory actions, offering a wide range of potential applications in tumor treatment, antibacterial therapy, wound healing, and bone tissue regeneration [20,21]. Our group previously developed a TA-loaded HPCH hydrogel [22]. In this hydrogel, TA serves as a cross-linking agent to enhance the mechanical properties of the hydrogel. Additionally, TA is slowly released from the hydrogel, thereby accelerating the healing of infected wounds.

This study hypothesized that the tannin-containing hydroxypropyl chitin hydrogel (HPCH/TA hydrogel) can be used as pulp capping agent and has the potential to control dental pulp inflammation. The objective of this study was to assess the effects of HPCH/TA hydrogel as a pulp capping material for vital pulp treatment in inflamed pulp.

## 2. Materials and Methods

### 2.1. Preparation of Hydrogels

HPCH was synthesized from chitin and propylene oxide using the method described by Ma et al. [22]. The freeze-dried HPCH was sterilized using ethylene oxide. To prepare the hydrogel, 30 mg of HPCH was dissolved in 1 mL of phosphate-buffered saline (PBS) at 4 °C. A total of 400 μL of TA solution (7.5 mg/mL) and 100 μL of FeCl_3_·6H_2_O solution (8 mg/mL) were filtered through a 0.22 μm membrane filter and added sequentially to the HPCH solution. The HPCH/TA hydrogels (with a final HPCH concentration of 2 wt%, TA concentration of 2 mg/mL, and FeCl_3_·6H_2_O concentration of 0.53 mg/mL) were obtained by warming to 37 °C. Control HPCH hydrogels (without TA) were prepared by adding a FeCl_3_·6H_2_O solution to the HPCH precursor solution.

#### 2.1.1. Temperature Sensitivity, Injectability, and Microstructure of the Hydrogels

The state of the hydrogels was observed at 4 °C and 37 °C, respectively. The injectability was measured by passing the solution through a 26 G microfine needle filled with the composite hydrogel solution. The composite hydrogels underwent freeze drying after being pre-cooled for 24 h at −80 °C. Scanning electron microscopy (SEM; tabletop microscope TM3030, Hitachi, Japan) was used to observe the microstructure after gold spraying.

#### 2.1.2. Mechanical Properties

Columnar hydrogels (diameter and height of 6 mm) were measured using an Instron universal testing machine (ElectroPlus E1000; Instron, Canton, MA, USA). The samples were kept at 37 °C to aid gelation. At a crosshead speed of 2 mm/min, the compressive modulus was determined by calculating the slope of the initial linear region within the strain range of 5% to 15%.

#### 2.1.3. Swelling Percentage

In order to ascertain the swelling ratio of the composite hydrogels, the solutions were cast in silicone molds with a diameter of approximately 10 mm and a depth of 5 mm prior to gelation. The samples were weighed after gelation (W_0_) and then immersed in 40 mL of PBS in an incubation shaker at 37 °C and 60 rpm. At specific time intervals, the samples were weighed (W_t_) after the excess PBS was removed with a tissue paper. The swelling ratio (%) was calculated using the following equation:

Swelling ratio (%) = (W_t_ − W_0_)/W_0_ × 100% (1)

#### 2.1.4. In Vitro Degradation Assay

The degradation property of the hydrogel was determined using a gravimetric method. In total, 1.5 g of the hydrogels was placed in PBS until it reached swelling equilibrium. The excess water was removed from the surface and the initial mass (W_0_) was recorded. The hydrogel was then placed in a 300-mesh nylon bag and immersed in 40 mL of PBS in an incubation shaker at 37 °C and 60 rpm. After that, the hydrogel was re-weighed (W_t_) at specific intervals following the absorption of excess water from the surface, and the remaining mass percentage was calculated with the following formula:

Relative weight (%) = (W_t_/W_0_) × 100%. (2)

#### 2.1.5. In Vitro Release of TA

About 1.5 g of HPCH/TA hydrogel was placed into 40 mL of PBS at 37 °C on a shaker set at 60 rpm. At specific time intervals, 1 mL of PBS was taken out for TA quantification, and an equal volume of fresh PBS was replenished. The concentration of TA released from the HPCH/TA hydrogel was measured using ultraviolet–visible (UV-Vis) spectrophotometry, and UV absorbance readings were taken at a wavelength of 278 nm. Quantitative analysis was carried out by comparing the results to previously prepared standard curves.

#### 2.1.6. Antibacterial Assay

Briefly, 200 μL of hydrogel was added to each well of a 24-well plate. Additionally, 200 μL of PBS was added to another set of wells as a control. Subsequently, 10 μL of a bacterial solution containing either *Streptococcus mutans* (*S. mutans*, ATCC 33342) or *Enterococcus faecalis* (*E. faecalis,* ATCC 29212) at a concentration of approximately 1 × 10^6^/mL was added to the respective wells. The plates were incubated for 24 h at 37 °C. *S. mutans* was cultured aerobically, while *E. faecalis* was cultured anaerobically. After the 24 h incubation period, 1 mL of PBS was added to each well to resuspend the surviving bacteria. A 10 μL aliquot of the resuspended bacterial solution was then taken and coated on BHI medium after gradient dilution. The number of colonies that had formed was then counted after 48 h.

### 2.2. Cell Culture

Ethical approval for this study was granted by the Ethics Committee of School and Hospital of Stomatology, Wuhan University (approval number WDKQ2024B48). This approval authorized the collection of human dental pulp cells (hDPCs) from healthy impacted third molars extracted from patients aged between 16 and 25 years. The collection of the third molars was carried out with the informed consent of the patients. The pulp tissue was extracted, cut into pieces of approximately 0.5 mm^3^, and placed into T25 culture flasks containing α-modified minimum essential medium (α-MEM; HyClone, Logan, UT, USA) with 20% fetal bovine serum (FBS; HyClone, Logan, UT, USA), 100 U/mL penicillin, and 100 μg/mL streptomycin. The pulp tissue was then cultured in a constant temperature incubator at 37 °C with 5% CO_2_. Once the cell clusters reached 80% growth confluence, they were digested and passaged onto 10 cm dishes for further cultivation in α-MEM with 10% FBS at 37 °C in a 5% CO_2_ incubator. Cells in passages 3–6 were used in this study.

### 2.3. Cell Viability Assay

After gelation at 37 °C, 100 µL of composite hydrogels was immersed in 1 mL of α-MEM and incubated at 37 °C in an incubator for 24 h. The supernatant was then extracted from the hydrogels and then taken for evaluation of the hydrogel’s biocompatibility. hDPCs were inoculated in 6-well plates at a density of 5 × 10^5^/well. When the cells reached around 90% confluence, they were exposed to HPCH hydrogel, HPCH/TA hydrogel, and iRoot BP Plus (commercial pulp capping material; Innovative Bioceramix, Vancouver, BC, Canada) extract liquid for 24 h, respectively. The cells were then stained with a live/dead cell staining kit (Calcein-AM/PI Double Staining Kit; Meilunbio, Dalian, China) in accordance with the instructions. The cells were incubated at 37 °C for 30 min and observed under a fluorescence microscope at 645 nm and 530 nm.

### 2.4. Cell Proliferation Assay

hDPCs were placed onto 96-well culture plates at a density of 5 × 10^3^ cells/well. They were then exposed to extract liquid of various materials: HPCH hydrogel, HPCH/TA hydrogel, and iRoot BP Plus. For each group, three sub-wells were used, and each well was treated with 100 µL of leaching solution for either 24 h or 72 h as per the instructions. Following the exposure, 10 µL of the cell counting kit-8 (CCK-8, Biosharp, Shanghai, China) was added to each well, and the plates were incubated under light protection for one hour. Finally, the absorbance at 450 nm was measured.

### 2.5. Quantitative Real-Time Polymerase Chain Reaction (qRT-PCR)

To produce inflamed human dental pulp cells (ihDPCs), hDPCs were treated with 1 μg/mL of lipopolysaccharide (LPS; Sigma Aldrich, St. Louis, MO, USA) [23]. The ihDPCs were then exposed to HPCH hydrogel, HPCH/TA hydrogel, and iRoot BP Plus extract liquid for either one hour or three hours. The levels of inflammatory cytokines were assessed using qRT-PCR. RNA extraction was carried out with TRIzol (Invitrogen, Carlsbad, CA, USA), followed by cDNA synthesis using a reverse transcription kit (Vazyme, Nanjing, China). The cDNA was then analyzed using a SYBR qPCR Master Mix detection kit (Vazyme, Nanjing, China). The qRT-PCR involved a 20 μL reaction mixture consisting of 2 μL cDNA and 200 nM of qRT-PCR primers. The primers, designed and synthesized by Bioengineering Biologicals (Shanghai Bioengineering, Shanghai, China), are listed in Table 1. The qRT-PCR analysis was performed with a Bio-Rad CFX96 real-time PCR system.

### 2.6. Enzyme-Linked Immunosorbent Assay (ELISA)

hDPCs were seeded in 6-well plates (5 × 10^4^/well), and when the cells grew to 80% fusion, LPS was added for treatment for three hours. iDPSCs were exposed to treatments with HPCH hydrogel, HPCH/TA hydrogel, and iRoot BP Plus extract liquid for a duration of 24 h. The concentrations of inflammatory factors interleukin-1β (IL-1β), interleukin-6 (IL-6), and tumor necrosis factor-α (TNF-α) in the supernatant were determined using ELISA kits (Elabscience Biotechnology Co., Ltd., Wuhan, China) according to the manufacturer’s instructions.

### 2.7. RNA Sequencing Analysis

Total RNA, isolated from ihDPCs (control, n = 3; HPCH/TA, n = 3) and hDPCs (NC, n = 3), was used for RNA sequencing (RNA-seq). The generated data were analyzed by Annoroad Gene Technology (Beijing, China). RNA was enriched and fragmented, followed by reverse transcription into cDNA. The cDNA library was prepared, size-selected, and PCR-amplified. RNA-seq was performed on the MGI platform, utilizing co-probe anchored polymerization technology for precise and efficient sequencing. Raw data were quality-filtered, aligned to the reference genome, and the gene expression levels were quantified. Differentially expressed genes (DEGs) between the two comparison combinations were performed using DESeq2 v1.18.1 software. The DEG screening criteria were as follows: |log_2_ Fold change| ≥ 1, *p*-value < 0.05. Kyoto Encyclopedia of Genes and Genomes pathway (KEGG) enrichment statistics of DEGs were achieved by clusterProfiler v4.6.2 software, and KEGG pathways with *p* < 0.05 were set as significant enrichment results.

### 2.8. Western Blot (WB) Analysis

hDPCs were seeded in 6-well plates (2 × 10^4^/well). Upon reaching 80% confluence, the cells were treated with LPS either alone or in combination with HPCH/TA hydrogel extract liquid or the NF-kappaB pathway inhibitor JSH-23 (MCE, Shanghai, China) for three hours. Cells were then lysed using a lysis buffer containing phosphatase inhibitors. The proteins were extracted and then quantified using the bicinchoninic acid protein assay (Thermo, Rockford, IL, USA). Approximately 40 μg of protein from each sample was separated on 10% SDS-PAGE gel and transferred to a polyvinylidene difluoride membrane at 100 V for 90 min. The membranes were then incubated in blocking buffer (Beyotime, Shanghai, China) for 30 min. The membranes were then incubated with the following primary antibodies at 4 °C overnight: NF-kappaB phospho-p65 (p-p65, 1:1000, Cat No. ET1604-27, HUABIO, Hangzhou, China), total-p65 (1:1000, Cat No. 80989-1-RR, Proteintech, Chicago, IL, USA), and β-actin (1:5000, Cat No. PMK058S, BIOPRIMACY, Wuhan, China). The membrane was then incubated with HRP-labeled Goat Anti-Rabbit IgG (H + L) for one hour at room temperature, and the bands were visualized using an Odyssey scanner (LI-COR, Lincoln, NE, USA). The target bands were quantified using Image J v1.53e software and normalized to β-actin. The original western blots can be found in Supplementary Materials.

### 2.9. Immunostaining

hDPCs were seeded onto cell slides in 12-well plates. Once the cells reached 40–50% confluence, they were treated as described above. The cells were then fixed in paraformaldehyde for 15 min. After fixation, they were blocked for 30 min at room temperature using a solution containing 3% sheep serum. Subsequently, the cells were left to incubate overnight at 4 °C with the following primary antibodies: p65 (1:200, Cat No. 80989-1-RR, Proteintech). The slides were then rinsed with PBS and incubated for one hour at 37 °C with Goat Anti-Rabbit IgG H&L (Alexa Fluor^®^ 488; antgene, Wuhan, China) secondary antibody. The cell nuclei were stained with 4’,6-diamidino-2-phenylindole (DAPI, Beyotime, Shanghai, China). Finally, images were captured using laser confocal microscopy (IX83 P2ZF, Olympus, Tokyo, Japan).

### 2.10. Induction of Rat Pulpitis and Direct Pulp Capping

This animal study was approved by the Experimental Animal Management and Use Committee of Wuhan University Animal Experiment Centre (approval number WP20230451). A total of 24 eight-week-old male Sprague Dawley rats (Vital River, Beijing, China) were used in the experiment. Pulpitis was induced under general anesthesia administered via intraperitoneal injection of 3% sodium pentobarbital. A total of 48 cavities were meticulously prepared on the occlusal surfaces of the maxillary right and left first molars using a 0.5 mm diameter 1/4 rounded carbide bur (MANI, Tokyo, Japan) under a stereomicroscope (Olympus, Tokyo, Japan). Following mechanical exposure of the pulp, 5 µL of LPS at a concentration of 10 mg/mL was applied to the cavities, as referenced in Minic et al. [24]. The cavities were then sealed with temporary filling material (Caviton; GC, Tokyo, Japan). After 24 h, the temporary filling material was carefully removed, and the cavities were thoroughly rinsed with 1% sodium hypochlorite. Bleeding was then controlled with sterile cotton pellets. The cavities were randomly divided into four groups: HPCH hydrogel group, HPCH/TA hydrogel group, iRoot BP Plus group, and blank control group (no capping material used) (n = 12/group). All cavities were restored using self-adhesive flowable resin composite (Dyad-flow, Kerr, Orange, CA, USA). The rats were sacrificed by CO_2_ inhalation at either four days (n = 6/group) or six weeks (n = 6/group). The bilateral maxillae were removed and preserved in 4% paraformaldehyde for 24 h.

### 2.11. Micro-Computed Tomography (micro-CT) Evaluation

The induced tertiary dentin in the 6-week group was analyzed using a micro-CT scanning scanner (Skyscan1276; Bruker, Kontich, Belgium). The micro-CT was operated with the following settings: source voltage, 85 kV; source current, 200 μA; pixel size, 6 μm. Following the scanning procedure, three-dimensional images of the specimens were reconstructed using NRecon v1.7.4.2 software (Bruker, Kontich, Belgium). The thickness of the formed dentin bridges beneath the capping material was evaluated using DataViewer v1.5.6.2 software (Bruker, Kontich, Belgium).

### 2.12. Histological Evaluation

The samples were placed in a 10% ethylene diamine tetraacetic acid solution for decalcification for 2–3 months. After the decalcification process was completed, the samples were embedded in paraffin wax and precisely sliced to a thickness of 5 µm using a paraffin slicer. Then, the sections were subjected to hematoxylin–eosin staining (H&E staining). The tissue sections of day-four samples were scored according to the scoring criteria for inflammatory cell response (Table 2) proposed by Louwakul et al. [25].

### 2.13. Immunohistochemistry

The sections were stained with IL-6 antibody using the immunohistochemistry method and viewed under light microscopy. After dewaxing and hydration, the tissue sections underwent antigen retrieval using pepsin. The sections were then incubated with the IL-6 primary antibody (1:200, Cat No. A21264, ABclonal, Wuhan, China) at 4 °C overnight. Subsequently, a biotin-labeled secondary antibody was added, followed by streptavidin–peroxidase for signal amplification. After DAB staining, the sections were observed under a microscope.

### 2.14. Statistical Analysis

All the in vitro experiments were conducted in triplicate and all quantitative data are presented as mean ± SD. SPSS 24.0 software (SPSS, Chicago, IL, USA) was used for statistical analysis of the data. Two-way analysis of variance (ANOVA) was performed for the CCK-8 assay. For other experiments that involve a single variable, one-way ANOVA was employed for statistical analysis. *p* values < 0.05 were considered statistically significant.

## 3. Results

### 3.1. Physicochemical Properties of the Hydrogels

**Figure 1:**
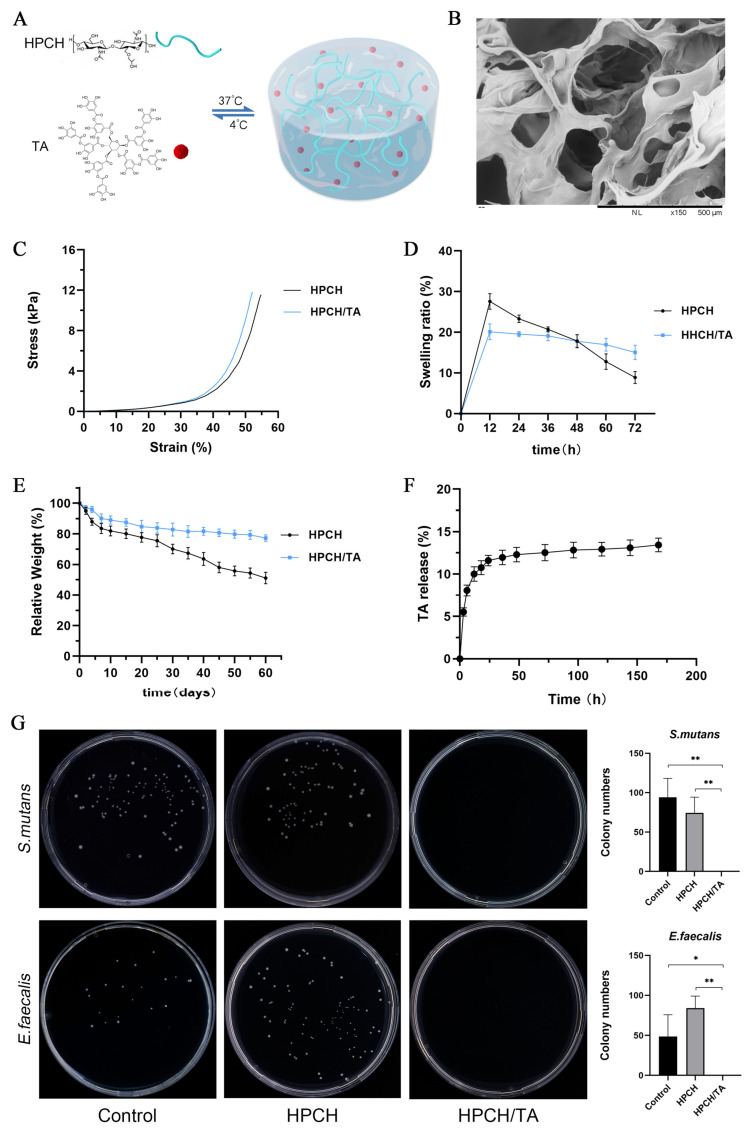
Characterization of the hydrogels. (**A**) Schematic structure of the HPCH/TA hydrogel. (**B**) Representative SEM images of HPCH/TA hydrogel. (**C**) Compressive stress–strain curves of HPCH hydrogel and HPCH/TA hydrogels. (**D**) Swelling behaviors of the above hydrogels. (**E**) In vitro degradation behaviors of the above hydrogels. (**F**) TA release curve of the HPCH/TA hydrogels. (**G**) No colony growth was observed in the presence of the HPCH/TA hydrogel, compared to the control group (PBS) and the HPCH hydrogel. Colony numbers analysis showed that the difference was significant. * *p* < 0.05 and ** *p* < 0.01.

A schematic illustration of the HPCH/TA hydrogel preparation process was presented in Figure 1A. The HPCH/TA mixtures (pre-gels) were translucent and slightly viscous in a liquid state at 4 °C (Figure S1A). At the physiological temperature of 37 °C, HPCH/TA demonstrates a gel state (Figure S1B). The injectability of the hydrogels was also demonstrated by injecting them through a syringe equipped with a 26G needle (Figure S1C,D). Scanning electron microscope (SEM) images show the porous structure of freeze-dried HPCH/TA hydrogels (Figure 1B). The stress–strain curve (Figure 1C) shows that the compression modulus of the HPCH hydrogel was 1.5 ± 0.15 kPa, and the compression modulus of the HPCH/TA hydrogel was 1.6 ± 0.17 kPa. All hydrogels demonstrated rapid liquid uptake, reaching equilibrium swelling within 24 h (Figure 1D). After 2 months, only 51.1% of HPCH remained in PBS, while 77.3% of HPCH/TA remained (Figure 1E). The release curve of TA (Figure 1F) exhibited an initial rapid release over the first 24 h, followed by a period of relative stability.

The antibacterial properties of the hydrogels against *S. mutans* and *E. faecalis* were shown in Figure 1G. After 24 h of co-culture, there was no bacterial growth observed in the HPCH/TA hydrogel. Data analysis showed that the HPCH/TA hydrogel was significantly more effective at inhibiting bacterial growth compared to the PBS control group and the HPCH hydrogel group.

### 3.2. The HPCH/TA Hydrogel Exhibited No Cytotoxicity and Effectively Promoted the Proliferation of hDPCs

**Figure 2:**
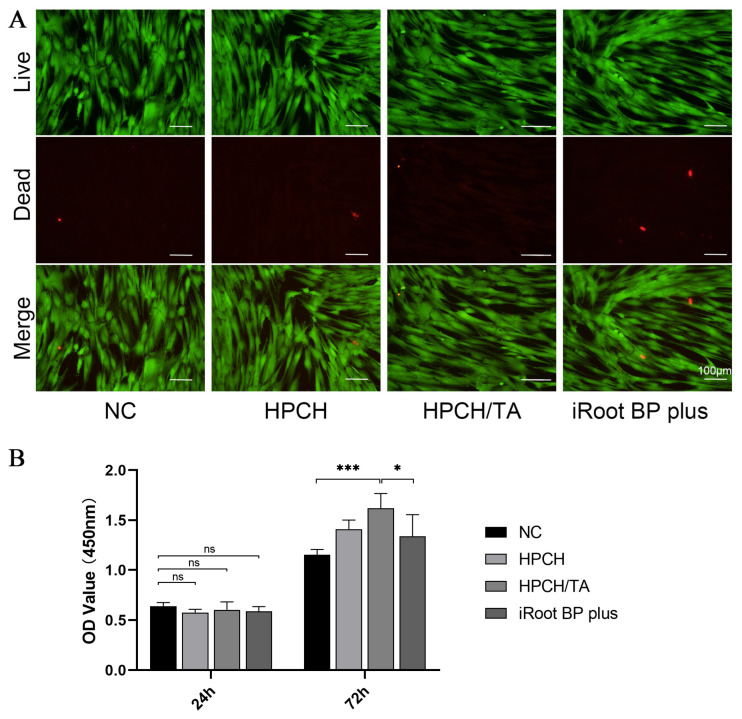
The HPCH/TA hydrogel exhibited excellent biocompatibility and effectively promoted the proliferation of hDPCs. (**A**) HDPCs stained with Calcein AM/PI double staining (live cell: green; dead cell: red). (**B**) HDPC proliferation was determined by the CCK-8 assay. * *p* < 0.05. *** *p* < 0.001. ns: *p* > 0.05.

The Live/Dead cell staining assay and CCK-8 assay were employed to examine the viability and proliferation of hDPCs in the hydrogels and iRoot BP Plus extract. Most viable cells (green fluorescence), with a minimal incidence of dead cells (red fluorescence), were observed in all hydrogel groups after 24 h of incubation (Figure 2A). The results suggested excellent biocompatibility of these hydrogels for biomedical applications. After 24 h, the cck-8 optical density (OD) values at 450 nm were not significantly different between groups (Figure 2B). However, after 72 h, the OD values were significantly higher for the HPCH/TA hydrogel compared to the control group (Figure 2B). These results showed that the HPCH/TA hydrogel effectively promoted the proliferation of hDPCs.

### 3.3. The HPCH/TA Hydrogel Suppressed the Expression of Inflammatory Cytokines in ihDPCs

**Figure 3:**
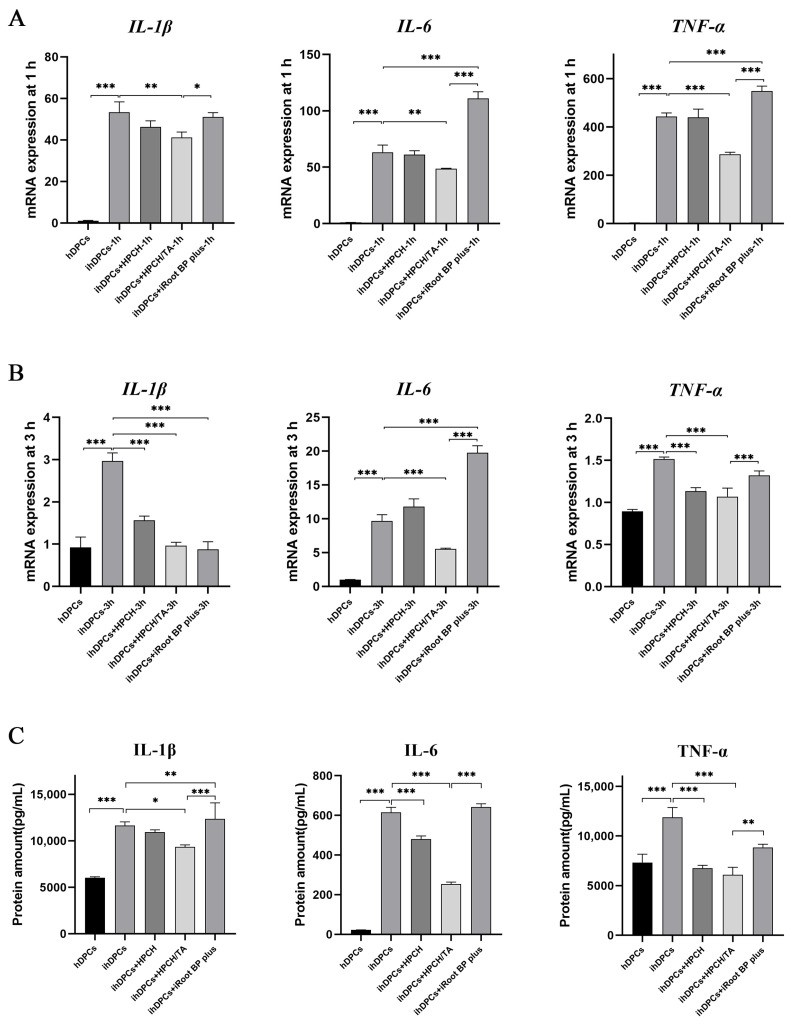
The effects of the hydrogels or iRoot BP Plus on the expression of inflammatory cytokines in ihDPCs. (**A**) The mRNA expression of the inflammatory cytokines *IL-1β*, *IL-6*, and *TNF-α* was evaluated after one hour of LPS stimulation following treatment with the hydrogels or iRoot BP Plus. (**B**) The mRNA expression of the above inflammatory cytokines was evaluated after three hours. (**C**) The protein levels of the inflammatory cytokines were evaluated after 24 h. * *p* < 0.05, ** *p* < 0.01, and *** *p* < 0.001.

The qRT-PCR and ELISA analyses were performed to evaluate the expression levels of inflammatory cytokines. The HPCH/TA hydrogel demonstrated a significant reduction in *IL-1β*, *IL-6*, and *TNF-α* mRNA expression in ihDPCs after one hour, in comparison to the control and iRoot BP Plus groups (Figure 3A). After a three-hour incubation period with the HPCH/TA hydrogel, there was also a notable decrease in *IL-1β*, *IL-6*, and *TNF-α* (Figure 3B).

The HPCH/TA hydrogel exhibited a significant reduction in the protein levels of IL-1β, IL-6, and TNF-α in ihDPCs after 24 h, compared to the LPS and iRoot BP Plus groups (Figure 3C). This reduction in protein levels aligned with the observed trend in mRNA variation. The findings indicated that the HPCH/TA hydrogel demonstrated effective anti-inflammatory properties in vitro.

### 3.4. Anti-Inflammatory Activity of the HPCH/TA Hydrogel May Occur through Inhibition of the NF-κB Signaling Pathway

**Figure 4:**
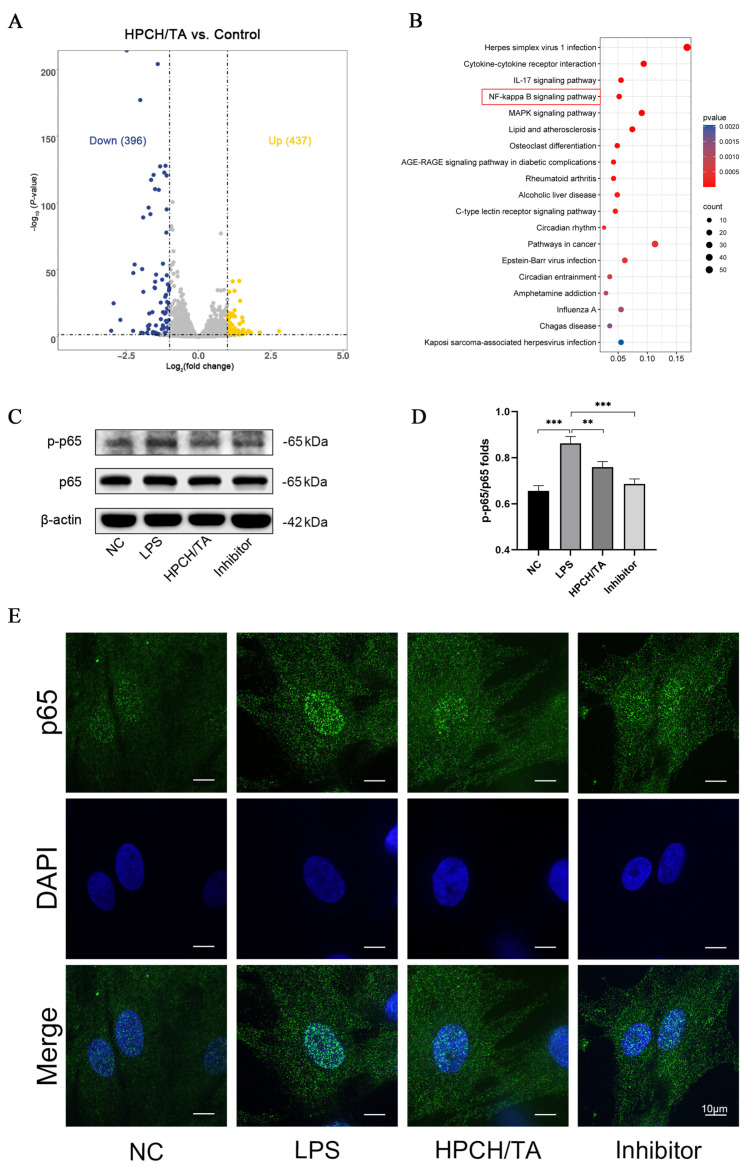
Anti-inflammatory activity of the HPCH/TA hydrogel may occur through inhibition of the NF-κB signaling pathway. (**A**) Volcano map showing differential genes (yellow plots represent differentially upregulated genes; blue plots are for differentially downregulated genes; gray plots represent the genes with no difference). (**B**) KEGG pathway enrichment analysis in DEGs. The red box showed a significant enrichment in pathways associated with the NF-κB pathway. (**C**) Expression of p65 and p-p65 was analyzed by Western blot. (**D**) The density of bands was analyzed using ImageJ software, and statistical analysis was performed to determine the ratio of p-p65 to total p65. (**E**) Immunofluorescence imaging of p65 in ihDPCs treated with LPS after three hours. The cells were stained with anti-p65 antibody (appearing in green) and counterstained with DAPI to identify cell nuclei (appearing in blue). ** *p* < 0.01 and *** *p* < 0.001.

To explore the underlying mechanism of the anti-inflammatory effects of HPCH/TA hydrogels, RNA-seq was performed. Based on the principal component analysis (PCA) of the different samples, there are significant differences between groups and little variation within groups (Figure S2A). In total, 883 DEGs were identified between the HPCH/TA hydrogel and control groups, with 396 downregulated and 437 upregulated (Figure 4A). Clustering of differentially expressed genes was visualized using heatmaps (Figure S2B). KEGG analysis of these genes revealed significant enrichment in the pathways of the NF-κB signaling pathway (Figure 4B).

The expression of p-p65 was upregulated following LPS stimulation, while JSH-23 (a specific inhibitor of NF-kappaB nuclear translocation) and HPCH/TA hydrogel exhibited suppressive effects on its expression (Figure 4C,D). Additionally, p65 translocated to the nucleus after three hours of LPS stimulation, and this process was inhibited by the inhibitor JSH-23. Cells treated with HPCH/TA hydrogel showed significantly reduced nuclear translocation of p65 compared to the LPS group (Figure 4E). These findings confirmed that the anti-inflammatory activity of the HPCH/TA hydrogel may occur through inhibition of the NF-κB signaling pathway.

### 3.5. The HPCH/TA Hydrogel Effectively Reduced Pulpal Inflammation and Promoted Reparative Dentin Formation in the Rat Model of Pulpitis

**Figure 5:**
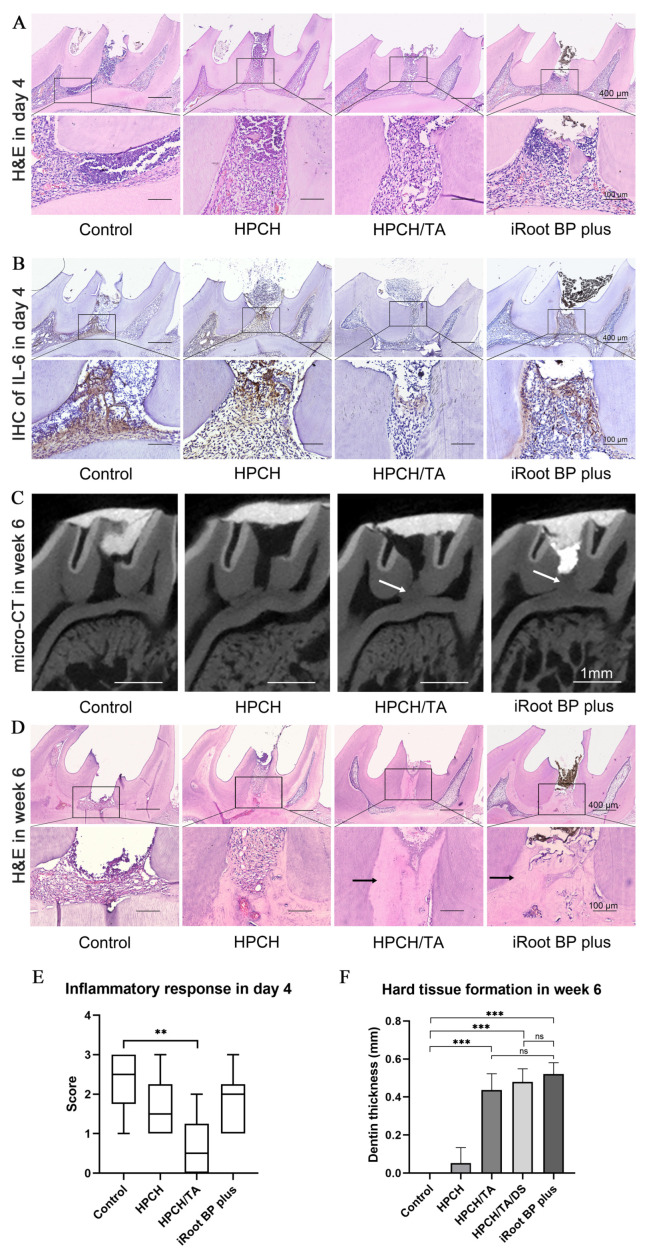
In vivo response to direct pulp capping with hydrogels and iRoot BP Plus. (**A**) H&E staining was used to detect inflammatory cell infiltration in the rat pulpitis model on day four after direct pulp capping with the hydrogels and iRoot BP Plus. (**B**) IL-6 expression was observed by immunohistochemical staining in the rat pulpitis model on day four. (**C**) Sagittal view micro-CT images of the surgical site from different samples at six weeks (arrow represents reparative dentine). (**D**) Observation of reparative dentin formation through H&E staining at six weeks (arrow represents reparative dentine). (**E**) The statistical analysis of inflammatory scores on day four. ** *p* < 0.01. (**F**) Statistical analysis of the relative thickness of the hard tissue formation based on micro-CT images. *** *p* < 0.001. ns: *p* > 0.05.

In the control group, severe inflammation with extensive necrosis was observed in the coronal and upper middle pulp tissues, along with significant infiltration of inflammatory cells towards the radicular pulp. The HPCH group showed a moderate accumulation of inflammatory cells at the injury site, with infiltration progressing towards the coronal pulp. On the other hand, the HPCH/TA groups exhibited few scattered inflammatory cells at the exposure site. In the iRoot BP Plus group, moderate inflammatory cell infiltration was observed under the capping material (Figure 5A). The HPCH/TA groups had notably lower inflammatory scores compared to the control group (Figure 5E). Additionally, the control group exhibited significantly higher IL-6 expression compared to the other treatment groups. The iRoot BP Plus and HPCH groups showed a moderate level of IL-6 expression, while the HPCH/TA groups expressed IL-6 at minimal levels at the exposed sites (Figure 5B). These findings confirmed that HPCH/TA effectively reduced pulpal inflammation in vivo.

After six weeks of capping, micro-CT imaging (Figure 5C) revealed the absence of hard tissue formation in the control group. In contrast, significant hard tissue formation was observed under the pulp capping material in the iRoot BP Plus group, which served as the positive control. Additionally, complete hard tissue formation was noted in both HPCH/TA groups. Hard tissue formation was observed in the HPCH group, but it was incomplete. The thickness of the hard tissue formation was similar between the HPCH/TA groups and the iRoot BP Plus group (Figure 5F). Representative H&E staining (Figure 5D) showed the absence of reparative dentin formation within the pulp cavity in the control group. Conversely, a complete reparative dentin formation was observed under the pulp capping material in the HPCH/TA and iRoot BP Plus groups.

## 4. Discussion

Innovative pulp capping materials are important for overcoming the limitations of traditional therapies in pulp–dentin regeneration. In this study, we combined TA and HPCH to create a new pulp capping biomaterial: HPCH/TA hydrogel. The inclusion of HPCH in pulp capping material provides both temperature sensitivity and injectability. The injectable hydrogel can be easily inserted into the pulp cavity and seamlessly adapt to cavity walls. Its temperature sensitivity allows the hydrogel to undergo in situ gelation at physiological temperatures, ensuring effective and uniform filling of irregular defects within the tooth structure [26]. The HPCH/TA hydrogel after gelatinization has sufficient structural strength to be used as a pulp capping material. Additionally, the porous structure of HPCH/TA hydrogel was confirmed. The porous structure makes it an ideal scaffold material that not only supports cell growth but also facilitates the delivery of bioactive molecules [27]. The results of our in vitro TA release assay showed that HPCH/TA hydrogels have the potential to serve as controlled release systems for TA, enabling localized and sustained delivery of the bioactive molecule. The initial burst release may be advantageous for the rapid onset of anti-inflammatory effects of TA, while the sustained release phase ensures prolonged exposure. The HPCH/TA hydrogel exhibits excellent physicochemical properties, making it an ideal choice for pulp capping material.

The HPCH/TA hydrogel effectively inhibited the growth of *S. mutans* and *E. faecalis*, suggesting its potential in preventing bacterial growth during the pulp capping process. In addition to its antibacterial properties, TA also demonstrated effective anti-inflammatory activity [22,28,29,30]. It has been demonstrated that TA exerts anti-inflammatory effects by inhibiting IL-6 and TNF-α production in stimulated macrophages [22,28,29]. In our study, the HPCH/TA hydrogel also suppressed the expression of inflammatory factors IL-1β, IL-6, and TNF-α in hDPCs. Furthermore, in the rat pulpitis model, the HPCH/TA hydrogel displayed significantly reduced inflammatory cell infiltration and IL-6 expression compared to the control group. These findings further confirmed the anti-inflammatory efficacy of TA on inflamed pulp tissue, providing new experimental evidence for its potential application in the treatment of pulpitis. 

To gain a deeper understanding of the anti-inflammatory effects, we explored the potential signaling pathways involved. In our study, RNA sequencing identified 883 DEGs between the HPCH/TA hydrogel and control groups. The KEGG analysis conducted on DEGs revealed significant enrichment within the pathways associated with the NF-κB signaling pathway. Previous research has established a link between the NF-κB pathway and inflammation induced by LPS [23,31]. NF-κB is a critical regulator in various immune pathways [32], inducing the expression of pro-inflammatory cytokines and regulating inflammasomes [33]. Our study not only confirmed the upregulation of pro-inflammatory cytokines after LPS stimulation but also demonstrated that treatment with the HPCH/TA hydrogel notably decreased their expression levels. Moreover, our findings revealed that HPCH/TA suppressed the phosphorylation of p65 and its translocation into the nucleus. These findings suggested that the HPCH/TA hydrogel may have therapeutic potential in alleviating LPS-induced inflammatory responses in hDPCs by inhibiting NF-κB signaling.

The dentin–pulp complex demonstrates remarkable regenerative potential after injury and infection [34]. Research indicates that resolving inflammation at relatively low levels of pro-inflammatory mediators can support tissue repair, highlighting the importance of controlling chronic inflammation to prevent inhibited repair mechanisms [35,36]. Notably, experiments have revealed that inhibiting LPS-induced NF-κB activity preserves the osteogenic capacity of dental pulp stem cells [37], suggesting the significant impact of controlling inflammation on reparative dentin formation in inflamed pulp. In our research, we found that the control and HPCH groups showed no significant reparative dentin formation. In contrast, the HPCH/TA group, which loaded with TA, displayed complete reparative dentin formation. Therefore, HPCH/TA as pulp capping material promoted reparative dentin formation by effectively controlling pulpal inflammation.

The clinical significance of our research lies in the pioneering application of the HPCH/TA hydrogel as a bioactive pulp capping material within vital pulp therapy, specifically targeting inflamed pulps. The hydrogel’s excellent anti-inflammatory property renders it invaluable in broadening the therapeutic indication of vital pulp therapy to encompass patients with inflammatory pulpal conditions. This material actively promoted the repair and regeneration of the inflamed pulp tissue by effectively mitigating inflammation and creating a conducive environment for healing. This represents a significant advancement over traditional pulp capping materials, which have limited anti-inflammatory effects. Furthermore, the injectable and temperature-sensitive properties of the HPCH hydrogel greatly facilitate the clinical procedure, further underlining its clinical value.

Although the HPCH/TA hydrogel offers many advantages, such as excellent anti-inflammatory properties and antibacterial capacity, its inherent limitations cannot be ignored. The transparent nature of the HPCH/TA hydrogel presents a challenge in practice, as it can make it difficult for dentists to differentiate and control the amount to be applied accurately. Furthermore, the HPCH/TA hydrogel lacks radiopacity, limiting the iconography to visualize and assess the site of the HPCH/TA hydrogel application through X-ray. Future research will modify the HPCH/TA hydrogel to enhance operational precision and imaging visibility by changing the color and radiopacity. Meanwhile, extensive preclinical studies on the HPCH/TA hydrogel are crucial to understand its anti-inflammatory, immunomodulation, and antibacterial properties. This will facilitate evaluating its efficacy and safety further onwards, ultimately promoting its potential as a bioactive pulp capping material for VPT.

## 5. Conclusions

The HPCH/TA hydrogel was temperature-sensitive, injectable, and possessed antibacterial and anti-inflammatory properties. It can suppress the expression of inflammatory cytokines in hDPCs, potentially by inhibiting the activity of the NF-κB pathway. It can effectively reduce pulpal inflammation and promote reparative dentin formation in a rat model of pulpitis. The HPCH/TA hydrogel may be a promising pulp capping material to promote inflamed pulp repair in vital pulp therapy. Further preclinical studies and well-designed clinical trials may provide more evidence to support the clinical use of HPCH/TA hydrogel in vital pulp therapy for pulpitis.

## Figures and Tables

**Table 1:** Primers used for qRT-PCR.

Gene	Sequence
*GAPDH*	Forward: GGCAAATTCCATGGCACCGT Reverse: TGGACTCCACGACGTACTCA
*IL-1β*	Forward: GCAGAAGTACCTGAGCTCGC Reverse: CATGGCCACAACAACTGACG
*IL-6*	Forward: TGCAATAACCACCCCTGACC Reverse: GTGCCCATGCTACATTTGCC
*TNF-α*	Forward: TCTTCTCGAACCCCGAGTGA Reverse: ATGAGGTACAGGCCCTCTGA

**Table 2:** Inflammatory cell response criteria [25].

Score	Definition
0	No inflammatory cell infiltration at or beneath the exposure site
1	Few scattered inflammatory cells at or beneath the exposure site (mild or slight response)
2	General or localized moderate inflammatory cell infiltration in the pulp proper at or beneath the exposure site (moderate response)
3	Severe inflammation and/or abscess formation at or beneath the exposure site (severe response)

## Data Availability

Dataset available on request from the authors.

## Supplementary Materials

The following supporting information can be downloaded at https://www.mdpi.com/article/10.3390/biom14091129/s1. Figure S1: Photographic illustration and characterization of the hydrogels. Figure S2: Transcriptome Differences Analyzed.
