# Supplementary material for: Injectable Tannin-Containing Hydroxypropyl Chitin Hydrogel as Novel Bioactive Pulp Capping Material Accelerates Repair of Inflamed Dental Pulp
Source: Biomolecules. 2024 Sep 6;14(9):1129. doi: 10.3390/biom14091129 (PMC11430630; doi:10.3390/biom14091129)
Supplement: Supplementary file 1 [file biomolecules-14-01129-s001.zip › supplementary materials.pdf]

Injectable Tannin-containing Hydroxypropyl Chitin Hydrogel as Novel Bioactive Pulp Capping Material Accelerates Repair of Inflamed Dental Pulp

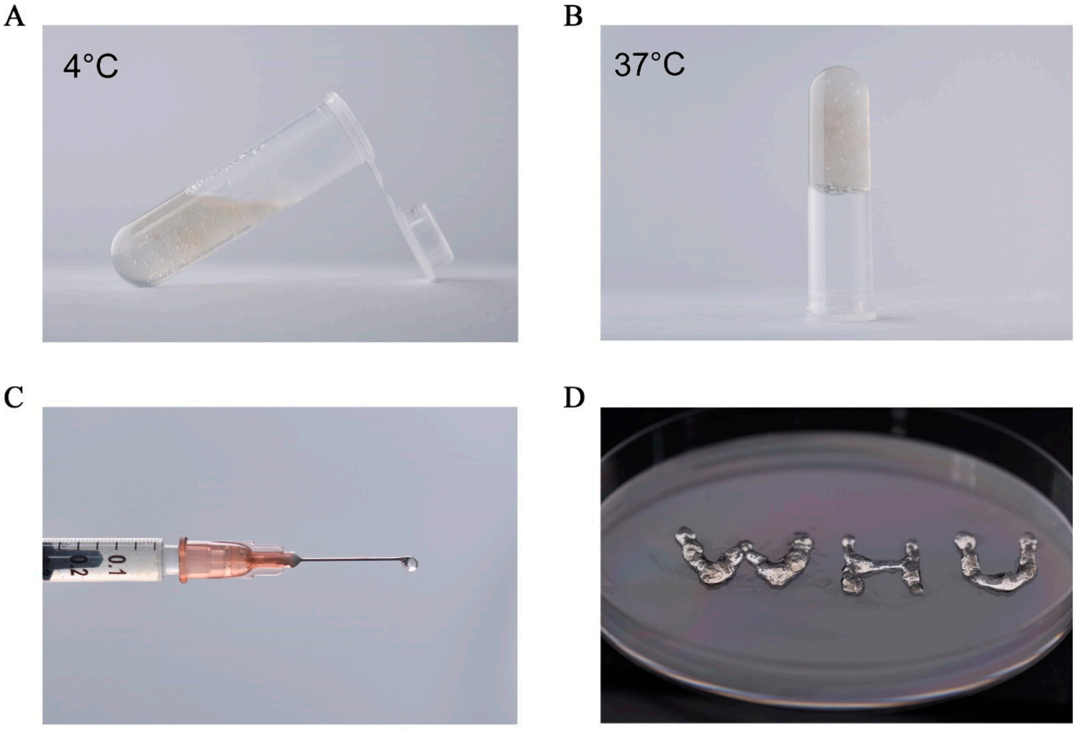

Figure S1. Photographic illustration and characterization of the hydrogels. (A) Photograph showing the HPCH/TA mixture solution at 4°C. (B) Photograph displaying the HPCH/TA hydrogel formed at 37°C. (C, D) Injectability of the HPCH/TA hydrogel through a 26G needle.

A

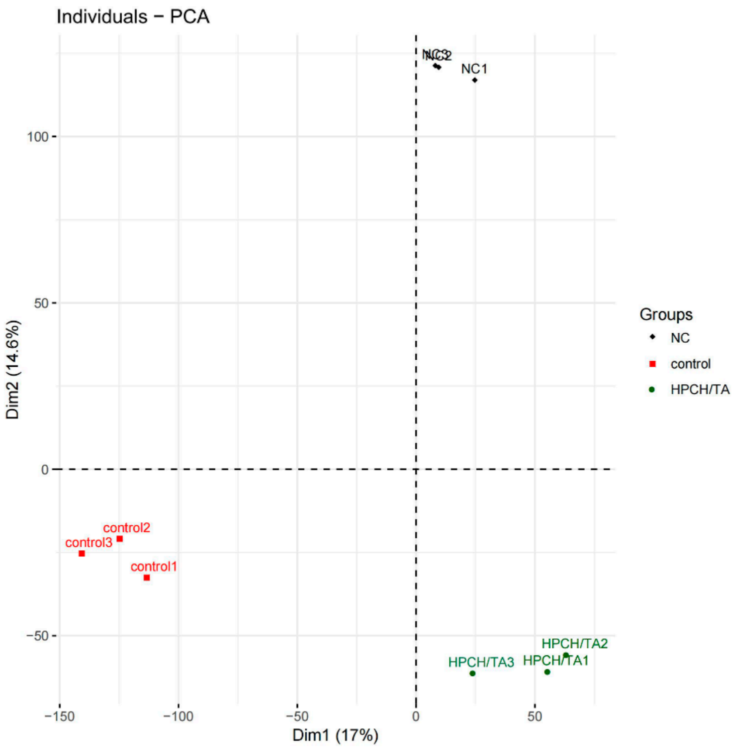

B

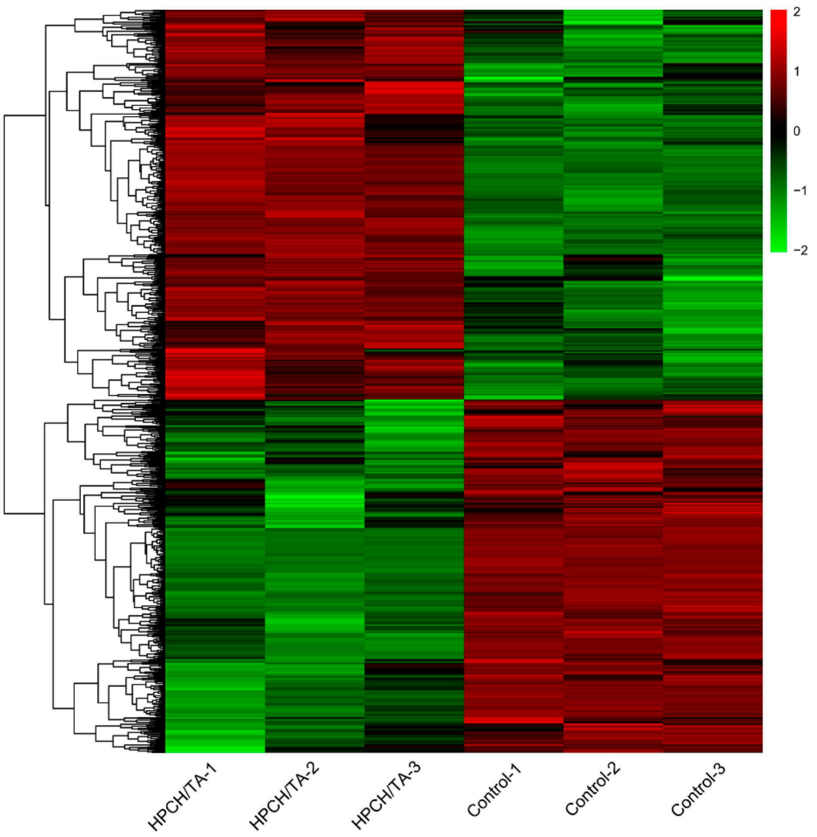

Figure S2. Transcriptome Differences Analyzed. (A) Principal component analysis (PCA) of transcriptome differences among three groups. (B) Clustering expression analysis of differentially expressed genes. The red colour indicated upregulated genes, while the green colour indicated downregulated genes.
