# Supplementary figures and images for: Injectable Tannin-Containing Hydroxypropyl Chitin Hydrogel as Novel Bioactive Pulp Capping Material Accelerates Repair of Inflamed Dental Pulp
Source: Biomolecules. 2024 Sep 6;14(9):1129. doi: 10.3390/biom14091129 (PMC11430630; doi:10.3390/biom14091129)

The original western blots of Figure 4C

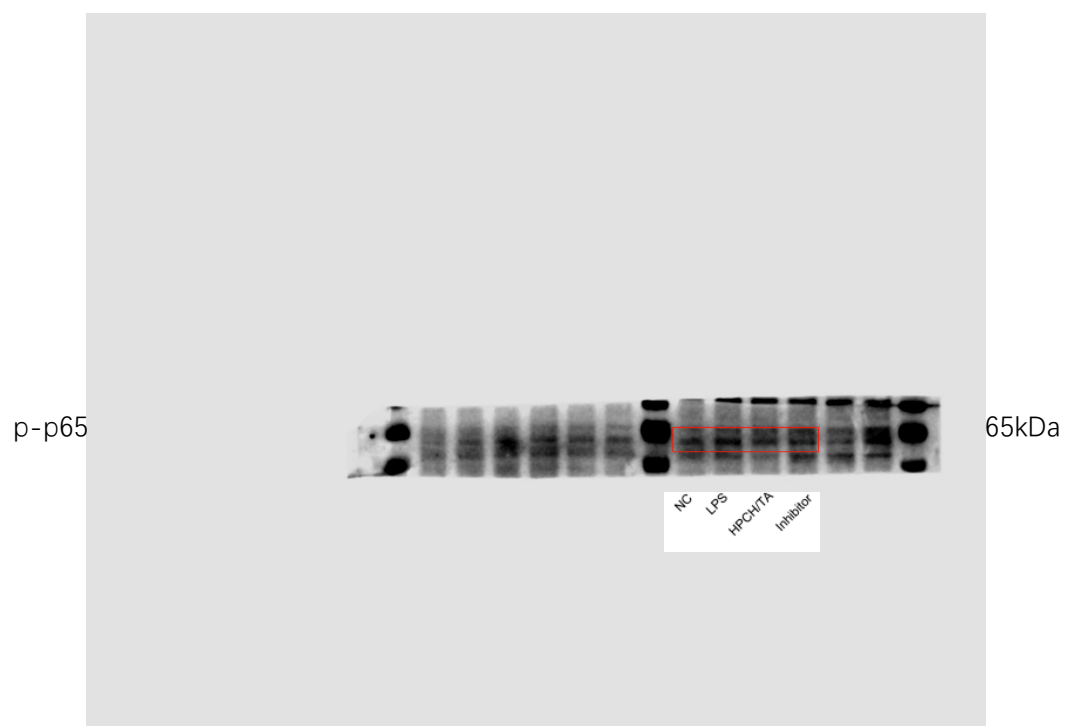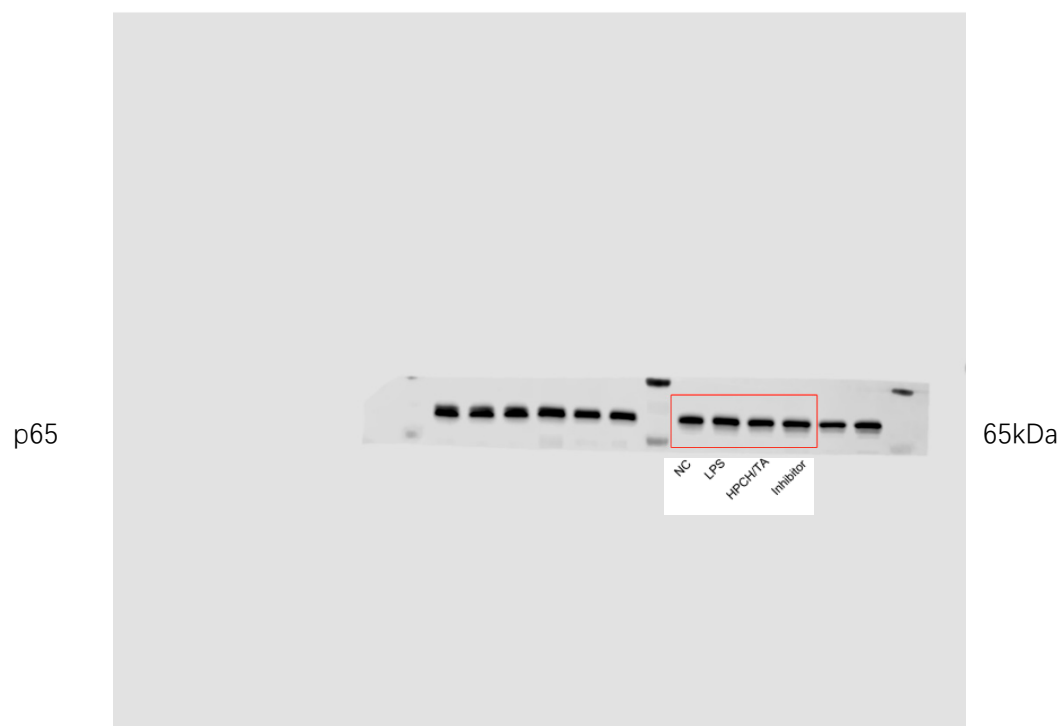

$\beta$ -actin

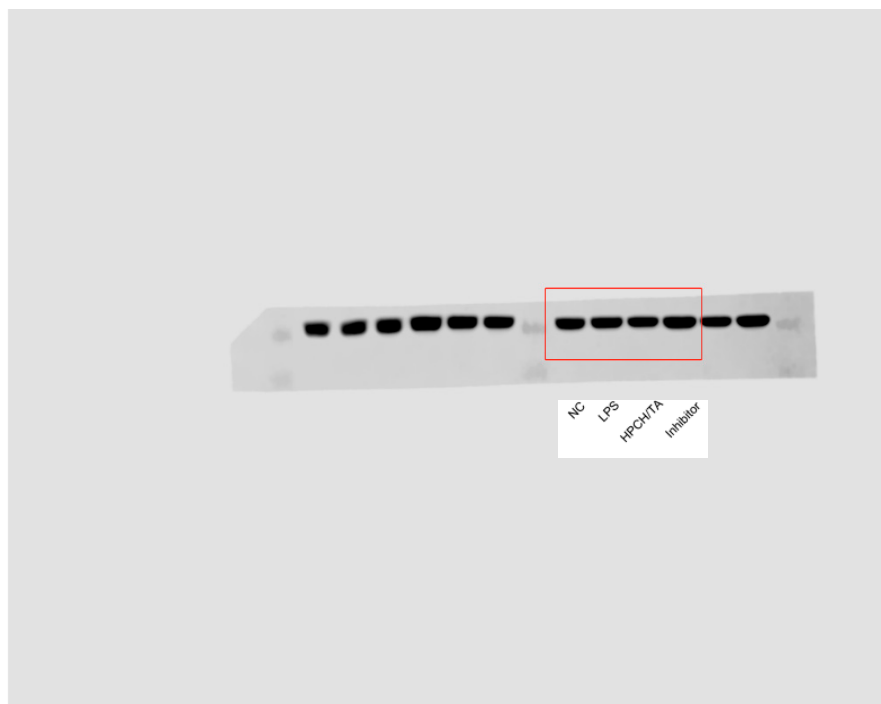

42kDa

Supplement: Supplementary file 1 [file biomolecules-14-01129-s001.zip › The original western blots of Figure 4C.pdf]
